# A third dose of the BNT162b2 mRNA vaccine sufficiently improves the neutralizing activity against SARS-CoV-2 variants in liver transplant recipients

**DOI:** 10.3389/fcimb.2023.1197349

**Published:** 2023-05-16

**Authors:** Takahiro Tomiyama, Rigel Suzuki, Noboru Harada, Tomokazu Tamura, Katsuya Toshida, Yukiko- Kosai-Fujimoto, Takahiro Tomino, Shohei Yoshiya, Yoshihiro Nagao, Kazuki Takeishi, Shinji Itoh, Nobuhiro Kobayashi, Hayato Ito, Sachiyo Yoshio, Tatsuya Kanto, Tomoharu Yoshizumi, Takasuke Fukuhara

**Affiliations:** ^1^ Department of Surgery and Sciences, Graduate School of Medical Sciences, Kyushu University, Fukuoka, Japan; ^2^ Department of Microbiology and Immunology, Faculty of Medicine, Hokkaido University, Sapporo, Japan; ^3^ Department of Liver Disease, The Research Center for Hepatitis and Immunology, National Center for Global Health and Medicine, Chiba, Japan

**Keywords:** SARS-CoV-2, liver transplantation, anti-SARS-CoV-2 vaccination, immunosuppressive treatment, mutant strains

## Abstract

**Introduction:**

We examined the neutralizing antibody production efficiency of the second and third severe acute respiratory syndrome coronavirus 2 (SARS-CoV-2) vaccine doses (2^nd^- and 3^rd^-dose) and neutralizing activity on mutant strains, including, the Ancestral, Beta and Omicron strains using green fluorescent protein-carrying recombinant SARS-CoV-2, in living-donor liver transplantation (LDLT) recipients.

**Methods:**

The patients who were administered vaccines other than Pfizer- BioNTechBNT162b2 and who had coronavirus disease 2019 in this study period were excluded. We enrolled 154 LDLT recipients and 50 healthy controls.

**Result:**

The median time were 21 days (between 1^st^ and 2^nd^ vaccination) and 244 days (between 2^nd^ and 3^rd^ vaccination). The median neutralizing antibody titer after 2^nd^-dose was lower in LDLT recipients than in controls (0.46 vs 1.00, P<0.0001). All controls had SARS-CoV-2 neutralizing antibodies, whereas 39 LDLT recipients (25.3%) had no neutralizing antibodies after 2^nd^-dose; age at vaccination, presence of ascites, multiple immunosuppressive treatments, and mycophenolate mofetil treatment were significant risk factors for nonresponder. The neutralizing activities of recipient sera were approximately 3-fold and 5-fold lower than those of control sera against the Ancestral and Beta strains, respectively. The median antibody titer after 3^rd^-dose was not significantly different between recipients and controls (1.02 vs 1.22, p=0.0758); only 5% recipients was non-responder. The neutralizing activity after third dose to Omicron strains were enhanced and had no significant difference between two groups.

**Conclusion:**

Only the 2nd-dose was not sufficiently effective in recipients; however, 3rd-dose had sufficient neutralizing activity against the mutant strain and was as effective as that in healthy controls.

## 1 Introduction

As of September 26, 2022, over 600 million people worldwide have been diagnosed with COVID-19, with over 6 million confirmed deaths. Severe acute respiratory syndrome coronavirus 2 (SARS-CoV-2) infection can cause serious complications and death, and a vaccine is required to prevent infection and severe disease. The infection rate was 3-times higher in solid organ transplant recipients than in non-solid organ transplant recipients, and the mortality rate was approximately twice as high in solid organ transplant recipients (Trapani et al., 2021). However, patient background and immunosuppressive drugs differ in each organ transplantation, and a more detailed organ-specific analysis is required in solid organ transplant recipients. Liver transplantation has a relatively good prognosis after COVID-19 compared with other organ transplantations, such as the heart and lungs; (Trapani et al., 2021) however, its mortality rate is higher than that of non-solid organ transplant patients (Becchetti et al., 2020). In liver transplant recipients, the risk of mortality from COVID-19 is generally driven by higher age and comorbidities (Choudhary et al., 2021).

Of the two major mRNA vaccines, Pfizer-BioNTechBNT162b2 and Moderna mRNA-1273, BNT162b2 was predominantly administered until the second administration in Japan. After the second vaccine dose, all healthy individuals acquired neutralizing antibodies against SARS-CoV-2 (Kageyama et al., 2021; Toniutto et al., 2022). In addition, vaccine-induced humoral and cell-mediated immunity reduces the risk of severe symptomatic SARS-CoV-2 related disease in immunocompetent patients (Khoury et al., 2021). However, immunosuppressive medication, age, glucocorticoid use, and alcohol consumption have been reported as risk factors for worsening of antibody titers after vaccination (Kageyama et al., 2021). In liver transplant (LT) recipients, the neutralizing antibody response to SASRS-CoV-2 after the second vaccine dose was weaker than in healthy controls (Toniutto et al., 2022). However, the efficacy of the third vaccine dose and changes in neutralizing activities against the mutant strain in LT recipients are still unknown. In this study, we examined the efficiency of neutralizing antibody production of the second and third SARS-CoV-2 vaccine doses and vaccine efficacy against mutant strains such as the Ancestral, Beta and Omicron strains in living-donor LT (LDLT) recipients.

## 2 Materials and methods

### 2.1 Study design and patients

We retrospectively reviewed data from 154 recipients who underwent LDLT at Kyushu University between January 1999 and November 2021. Patients with COVID-19 before the study were excluded. As a control, we retrospectively reviewed data from 31 LDLT donors who underwent liver resection at Kyushu University between January 2008 and November 2021 and 19 healthy volunteers who were clinical staff at Kyushu University. We collected sera from recipients and controls after the second and third doses of the SARS-CoV-2 vaccine. Patients who received only Pfizer-BioNTechBNT162b2 for the first, second, and third time were included in this study. Patients who had COVID-19 during the study period were excluded. In Japan, it was recommended that the second vaccine should be taken 21 days or later after the first vaccination and that the third vaccine should be taken at about six months after the second vaccination. The timing of vaccination of patients in this study was optional. The median time between first and second vaccination was 21 days (range: 21-36 days), and the median time between second and third vaccination was 244 days (range: 160-276 days). Since it takes approximately one week for antibody production after vaccination (Dagan et al., 2021), blood samples were taken when patients visited our hospital one week or later after the vaccine was administered. The median time from the second and third vaccinations to serum collection were 83 (range: 7–261 days) and 95.5 (range: 14-159 days) days, respectively. The study protocol was approved by the Institutional Review Board of Kyushu University Hospital (approval number 2020-639). This study was conducted in accordance with the Declaration of Helsinki, 1996. An optout approach was employed to obtain informed consent from our patients and personal information was protected during data collection.

### 2.2 Enzyme-linked immunosorbent assay

Serum SARS-CoV-2 IgG antibody levels were measured using an ELISA kit (E-EL-E-602, Elabscience, Houston, Texas, USA) according to the manufacturer’s instructions. A total of 100 μL serum samples were diluted 1:20 in sample dilution buffer and incubated at 37°C for 45 min. After washing, 100 μL horseradish peroxidase-conjugated receptor-binding domain antigen working solution was added, and the mixtures were incubated at 37°C for 30 min. After washing, 90 μL of substrate reagent was added, and the mixtures were incubated at 37°C for 15 min. Fifty μL stop solution was added, and the plate was read immediately at 450 nm. The cut-off value was calculated as 0.13 + negative control. The patients, whose antibody titers were below the cut-off value, were defined as non-responder. It is necessary to correct for the difference in absorbance between wells in order to compare antibody titer in each well, because this kit was originally a qualitative detection one. Hence, we divided the absorbance by positive control, and calculated comparative absorbance values.

### 2.3 Cell culture

TMPRSS2-expressing Vero E6 (VeroE6/TMPRSS2) cells were obtained from the Japanese Collection of Research Bioresources Cell Bank (JCRB1819) and maintained in low-glucose Dulbecco’s modified Eagle’s medium (DMEM; Sigma-Aldrich, St. Louis, MO, USA) with 10% fetal bovine serum (FBS) (Biowest, Bradenton, France) and G418 (Nacalai Tesque, Kyoto, Japan). HEK293-C34 cells were gifted by Y Matsuura at Osaka University and maintained in high-glucose DMEM (Nacalai Tesque) with 10% FBS and 10 μg/ml blasticidin (solution) (InvivoGen, California, USA), and the exogenous expression of ACE2 and TMPRSS2 was induced by the addition of doxycycline hydrochloride (1 μg/ml) (Sigma-Aldrich). All the above cells were cultured at 37°C under 5% CO_2_.

### 2.4 SARS-CoV-2 reverse genetics

Recombinant SARS-CoV-2 was generated by circular polymerase extension reaction (CPER) as previously described (Torii et al., 2021). Briefly, nine DNA fragments encoding the partial genome of SARS-CoV-2 (strain WK-521, PANGO lineage A; GISAID ID: EPI_ISL_408667) were prepared by PCR using PrimeSTAR GXL DNA polymerase (Takara). A linker fragment encoding the hepatitis delta virus ribozyme, bovine growth hormone poly A signal, and cytomegalovirus promoter was also prepared using PCR. The corresponding SARS-CoV-2 genomic regions, PCR templates, and primers used for this procedure are summarized in **Table S1**. Ten DNA fragments were mixed and used for CPER (Torii et al., 2021). To prepare green fluorescent protein (GFP)-expressing replication-competent recombinant SARS-CoV-2, we used fragment 9, in which the *GFP* gene was inserted into the *ORF7a* frame instead of the authentic F9 fragment (**Table S1**) (Torii et al., 2021).

To prepare rBeta S-GFP, the fragment of the viral genome corresponding to the region of fragment 8 (**Table S1**) was subcloned from a Beta isolate (strain hCoV-19/Japan/TY8-612-P1/2021; GISAID ID: EPI_ISL_1123289). rBA.1 S-GFP was gifted from K Sato at Tokyo University (Yamasoba et al., 2022). Nucleotide sequences were determined using a DNA sequencing service (Fasmac), and the sequence data were analyzed using ApE.

To produce recombinant SARS-CoV-2 (seed viruses), CPER products were transfected into HEK293-C34 cells using TransIT-LT1 (Takara) according to the manufacturer’s protocol. One day post-transfection, the culture medium was replaced with high-glucose DMEM (Nacalai Tesque) containing 2% FBS, 1% PS, and 1μg/ml doxycycline. At 6–10 days post-transfection, the culture medium was harvested and centrifuged, and supernatants were collected as seed viruses.

### 2.5 SARS-CoV-2 preparation and titration

The chimeric recombinant SARS-CoV-2 [rB.1.1 S-GFP (Ancestral), rBeta S-GFP(Beta), and rBA.1 S-GFP(Omicron)] (**Figure S1A**) were amplified in Vero E6/TMPRSS2 cells, and the culture supernatants were harvested and stored at −80°C until use. Infectious titers in the culture supernatants were determined using 50% tissue culture infective doses (TCID_50_). The culture supernatants of cells were inoculated onto VeroE6/TMPRSS2 cells in 96-well plates after serial 10-fold dilution with low-glucose DMEM containing 2% FBS and 1 mg/ml G418, and the infectious titers were determined 96 h post-infection (hpi). All experiments involving SARS-CoV-2 were performed in biosafety level-3 laboratories following standard biosafety protocols approved by Hokkaido University.

### 2.6 Neutralizing antibody titer assay

7.5×10^3^ VeroE6/TMPRSS2 cells per well were seeded in 96-well plates and maintained in high-glucose DMEM containing 10% FBS and 1% PS. The cells were then incubated overnight. The next day, each serum was serially diluted 3-fold in the culture medium with a first dilution of 1:10 (final dilution range of 1:10 to 1:21,870). The diluted serum was incubated with 140 TCID_50_ of the chimeric recombinant SARS-CoV-2 at 37°C for 1 h. Next, the mixture of chimeric recombinant SARS-CoV-2 and serum was added to VeroE6/TMPRSS2 cells in the 96-well plate. At 1 hpi, the cells were washed and replaced with high-glucose DMEM containing 10% FBS and 1% PS.

GFP fluorescence was detected using the ECLIPSE Ts2 (Nikon) after incubating the plates at 37°C for 34–36 h. Then, the luminance of GFP was calculated using Image J. A GFP signal with a luminance value >150 in one field of view was considered positive (**Figure S1B**). The neutralizing antibody titer was defined as the minimum serum dilution at which the GFP signal was positive. The neutralization titer of each serum sample was determined using duplicate assays. Data were initially plotted using GraphPad Prism 9 software (GraphPad Software).

For RT-qPCR, after incubating the plates at 37°C for 34–36 h, the viral RNA was extracted from the supernatants using the PureLink^®^ RNA Mini Kit (Invitrogen). The sample was used as a template for RT-qPCR performed according to the manufacturer’s protocol using the One Step PrimeScript™ III RT-qPCR Mix (Takara), primers and probe (see **Table S1**). Fluorescent signals were acquired using the StepOnePlus™ Real-Time PCR System (Applied Biosystems). The assay for each serum sample was performed in duplicate, and the 50% neutralization titer (NT_50_) was calculated using Prism 9 software.

### 2.7 Statistical analysis

Categorical variables, presented as numbers and percentages, were compared using Pearson’s chi-square test. Based on their distributions, continuous variables were presented as medians with ranges and compared using Student’s *t*-test. Any variable in the univariate analysis identified as significant (*p*<0.05) was considered a candidate for the multivariate logistic regression. Ineffectiveness against SARS-CoV-2 vaccination examined by ELISA was used to establish a univariate and multivariate logistic regression model. All statistical analyses were performed using JMP Pro 15 software (SAS Institute, NC, USA) and R software version 3.6.2.

### 2.8 Patient characteristics

The median control age of the controls and LDLT recipients were 37 (range: 21–60) and 66 (range: 29–84 years) years, respectively. The number of males in the control group was 33 (64.7%). The median duration between vaccination and antibody measurement in the control and LDLT groups were 95.5 (range: 8–261 days) and 78.5 (range: 7–205) days, respectively. Patient characteristics of the LDLT recipients are shown in **Table 1**. The LDLT recipients were older and more frequently female than the controls (p<0.0001 and p=0.0172, respectively). The duration between vaccination and antibody measurement was shorter in the LDLT recipients than in the controls (p<0.0001).

**Table 1:** Patient characteristics in LDLT recipients.

Variable	Median (range) or Number (%)
Age at vaccine (year range)	66 (29-84)
Sex (male)	70 (45.4%)
Blood type-incompatibility	22 (13.8%)
Days between vaccination and antibody measurement	78.5 (7-205)
Years between LDLT and vaccination	8.75 (0.23-22.6)
Etiology: Hepatocellular disease/ Cholestatic disease/ others	109/30/15 (70.8/19.5/9.7%)
History of HCC	66 (42.8%)
HCC at Vaccination	0 (0%)
Hypertension	45 (29.2%)
Diabetes mellitus	43 (27.9%)
HgbA1c	6.7 (5.9-8.6)
Dyslipidaemia	20 (13.0%)
Presence of esophageal varices at vaccination	15 (9.7%)
Presence of ascites at vaccination	5 (3.2%)
IS treatment	
Tacrolimus	105 (68.1%)
Cyclosporine	32 (20.8%)
Everolimus	23 (14.9%)
MMF	74 (48.1%)
Prednisone	42 (27.2%)
Number of IS treatment (1/2/3)	56/74/24 (36.3/48.1/15.6%)
Amount of IS treatment	
Tacrolimus (mg)	2 (0.5-8)
Cyclosporine (mg)	75 (25-100)
Everolimus (mg)	1 (0.5-1.5)
MMF (mg)	1000 (500-2000)
Prednisone (mg)	5 (1-15)
Serum IS treatment level	
Tacrolimus (ng/ml)	4 (0.52-21.3)
Cyclosporine (ng/ml)	72.3 (19.5-199)
Everolimus (ng/ml)	(0-10.8)
Hemoglobin (g/dl)	13.5 (8.7-17.0)
Hematocrit (%)	41.2 (29-53.4)
Neutrophils (n/μl)	3569 (1124-10908)
Lymphocytes (n/μl)	2257 (403-5563)
Albumin (g/dl)	4.1 (2.9-5.0)
Total bilirubin (mg/dl)	0.8 (0.2-4.4)
eGFR (ml/min/1.73m^2^)	60.9 (7.6-178.3)
AST (IU/ml)	23 (8-201)
ALT (IU/ml)	18 (3-163)

Data are presented as median (range) or n (%).

HCC, hepatocellular carcinoma; MMF, mycophenolate mofetil; eGFR, estimated.

## 3 Results

### 3.1 Anti-SARS-CoV-2 IgG antibody production by SARS-CoV-2 vaccination in LDLT recipients

**Figure 1:**
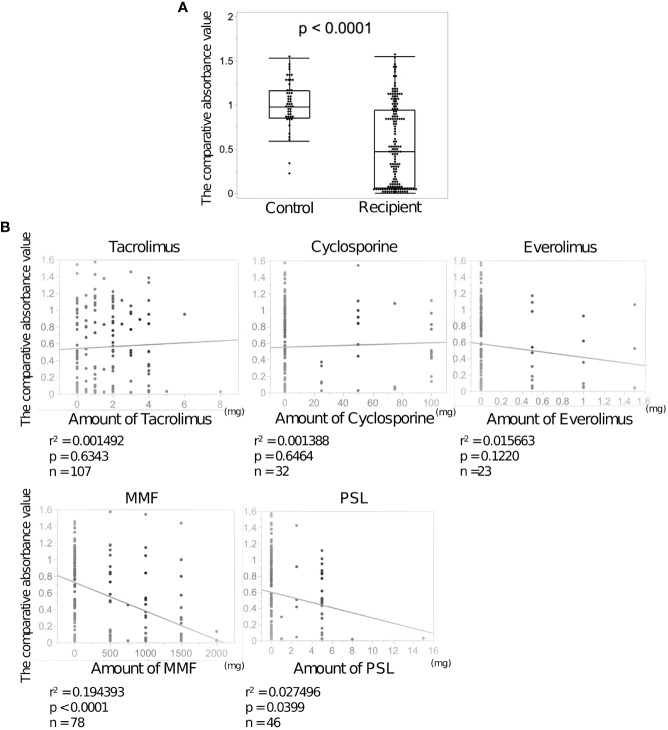
Anti-SARS-CoV-2 spike protein antibodies after the second dose of BNT162b2 vaccine in the sera of living donor liver transplantation (LDLT) recipients and healthy controls. **(A)** The anti-SARS-CoV-2 spike protein antibodies in LDLT recipients and healthy controls were measured using enzyme-linked immunosorbent assay (ELISA). **(B)** Correlation between each immunosuppressant dose and neutralizing antibody concentration.

SARS-CoV-2 IgG antibody titers in LDLT recipient and control sera were determined using ELISA. The median neutralizing antibody titer was lower in the recipients than in the controls (0.46 vs 1.00, p<0.0001, **Figure 1A**). Neutralizing antibodies were induced in all controls by the second vaccine dose, while 39 LDLT recipients (25.3%) had no neutralizing antibodies. We compared the characteristics of LDLT recipients with and without induced antibody production (responders and non-responders, respectively) (**Table 2**). Non-responders were older and had higher rates of ABO incompatibility, Hepatocellular carcinoma (HCC) history, and mycophenolate mofetil (MMF) treatment, and higher amount of ascites than responders. The number of immunosuppressant therapies is higher in non-responders than in responders. **Figure 1B** shows the association of various immunosuppressant doses with the level of antibody titers. Multivariate analysis identified age at vaccination, presence of ascites at vaccination, multiple immunosuppressive treatments, and MMF treatment as the factors involved in non-response to the second vaccine dose (**Table 3**). Additionally, we investigated the correlation between the spike protein IgG antibody titer and the dose of each immunosuppressants and found that the IgG antibody titer was significantly lower in patients receiving a high MMF and prednisolone dose (**Figure 1B**).

**Table 2:** Difference between patient characteristics in LDLT recipients.

Variable	Responder (n=115)	Non-responder (n=39)	p-value
Age at vaccine (year range)	66 (29-83)	68 (39-84)	**0.0439**
Sex (male)	55 (47.8%)	15 (38.5%)	0.3101
Blood type-incompatibility	11 (9.6%)	10 (25.6%)	**0.0115**
Days between vaccination and antibody measurement	76 (7-205)	83 (18-123)	0.9702
Years between LDLT and vaccination	9.5 (0.23-22.6)	7.4 (0.24-20.8)	0.0694
Etiology: Hepatocellular disease/ Cholestatic disease/ others	83/21/11 (72.1/18.3/9.6%)	26/9/4 (66.7/23.1/10.3%)	0.7843
History of HCC	55 (47.8%)	11 (28.2%)	**0.0324**
Hypertension	32 (27.8%)	13 (33.3%)	0.5134
Diabetes mellitus	32 (27.8%)	11 (28.2%)	0.9636
HgbA1c	6.8 (6.0-8.6)	6.6 (5.9-7.7)	0.2561
Dyslipidaemia	14 (12.2%)	6 (15.4%)	0.6062
Presence of esophageal varices at vaccination	12 (10.4%)	3 (7.7%)	0.6177
Presence of ascites at vaccination	1 (0.9%)	4 (10.3%)	**0.0148**
IS treatment			
Tacrolimus	80 (69.6%)	25 (64.1%)	0.5268
Cyclosporine	26 (22.6%)	6 (15.4%)	0.3366
Everolimus	16 (13.9%)	7 (18.0%)	0.5412
MMF	42 (36.5%)	32 (82.1%)	**<0.0001**
Prednisone	28 (24.4%)	14 (35.9%)	0.1617
Number of IS treatment (1/2/3)	50/53/12 (43.4/46.1/10.4%)	6/21/12 (15.4/53.9/30.8%)	**0.0007**
Amount of IS treatment			
Tacrolimus (mg)	2 (0.5-6)	2 (0.5-8)	0.6680
Cyclosporine (mg)	87.5 (25-100)	62.5 (25-75)	0.1476
Everolimus (mg)	0.5 (0.5-1.5)	1 (0.5-1.5)	0.0919
MMF (mg)	1000 (500-2000)	1000 (500-2000)	0.0534
Prednisone (mg)	5 (1-5)	5 (1-15)	0.9724
Serum IS treatment level			
Tacrolimus (ng/ml)	4 (0-21.3)	5.9 (0.8-16.5)	**0.0495**
Cyclosporine (ng/ml)	67 (20-199)	117 (31-185)	0.2093
Everolimus (ng/ml)	2.6 (0-10.8)	5 (0-7.2)	0.3666
Hemoglobin (g/dl)	13.6 (8.7-17.0)	13.0 (9.4-15.8)	0.0572
Hematocrit (%)	41.6 (29.0-53.4)	40.8 (30.8-49.7)	0.3268
Neutrophils (n/μl)	3561 (1128-9558)	3579 (1488-10908)	0.9618
Lymphocytes (n/μl)	2330 (404-5563)	1677 (496-4849)	**0.0027**
Albumin (g/dl)	4 (2.9-5.0)	4.1 (3.1-4.7)	0.4990
Total bilirubin (mg/dl)	0.8 (0.2-4.4)	0.8 (0.3-3.7)	0.5925
eGFR (ml/min/1.73m^2^)	62.1 (7.9-178.3)	51.9 (7.6-104.6)	**0.0113**
AST (IU/ml)	23 (8-136)	23 (13-201)	**0.0098**
ALT (IU/ml)	19 (3-142)	15 (4-163)	**0.0387**

**Table 3:** Predictors of non-responder.

Variables	Univariate analysis			Multivariate analysis		
	OR	95%CI	P-value	OR	95%CI	P-value
Age at vaccination	1.04	1.00-1.09	0.0494	1.10	1.02-1.19	0.0090
Sex (Male)	0.68	0.32-1.43	0.3115			
Years between LDLT and vaccination (year)	1.04	0.88-1.01	0.0901			
Days between vaccination and antibody measurement	0.99	0.99-1.00	0.6690			
Etiology: Hepatocellular disease / Cholestatic disease Hepatocellular disease / others Cholestatic disease / others	0.73 0.86 1.18	0.30-1.79 0.25-2.93 0.29-4.71	0.4932 0.8116 0.8162			
Hypertension (yes)	1.30	0.59-2.81	0.5169			
Diabetes mellitus (yes)	1.02	0.45-2.29	0.9637			
HgbA1c	0.50	0.0004-5.3	0.2766			
Dyslipidaemia (yes)	1.31	0.47-3.69	0.6070			
Presence of esophageal varices at vaccination	0.72	0.19-2.68	0.6190			
Presence of ascites at vaccination	13.0	1.40-120	0.0237	30.9	1.20-801	0.0387
Any triple and double/single IS treatment	4.23	1.64-10.9	0.0028	3.61	1.03-12.7	0.0455
Amount of IS treatment						
Tacrolimus (mg/day)	1.04	0.82-1.32	0.7382			
Cyclosporine (mg/day)	0.99	0.97-1.00	0.1414			
Everolimus (mg/day)	1.96	0.75-5.15	0.1819			
MMF (mg/day)	1.00	1.00-1.00	<0.0001	1.00	1.00-1.00	0.0001
Prednisone (mg/day)	1.12	0.97-1.30	0.1223			
Serum IS treatment level						
Tacrolimus (ng/ml)	1.08	0.98-1.20	0.1295			
Cyclosporine (ng/ml)	1.00	0.99-1.01	0.9311			
Everolimus (ng/ml)	1.09	0.89-1.34	0.3989			
Hemoglobin (g/dl)	0.80	0.65-0.98	0.0290	0.15	0.015-1.60	0.1111
Hematocrit (%)	0.96	0.89-1.03	0.2369			
Neutrophils (n/μl)	1.05	1.01-1.08	0.0084	0.99	0.86-1.13	0.8294
Lymphocytes (n/μl)	0.95	0.91-0.98	0.0044	0.94	0.81-1.10	0.4640
Albumin (g/dl)	0.78	0.31-1.96	0.5952			
Total bilirubin (mg/dl)	0.91	0.43-1.93	0.8120			
eGFR (ml/min/1.73m^2^)	0.97	0.96-0.99	0.0044	0.98	0.96-1.01	0.1613

### 3.2 Neutralizing activity of sera against SARS-CoV-2 variants after second vaccine dose

**Figure 2:**
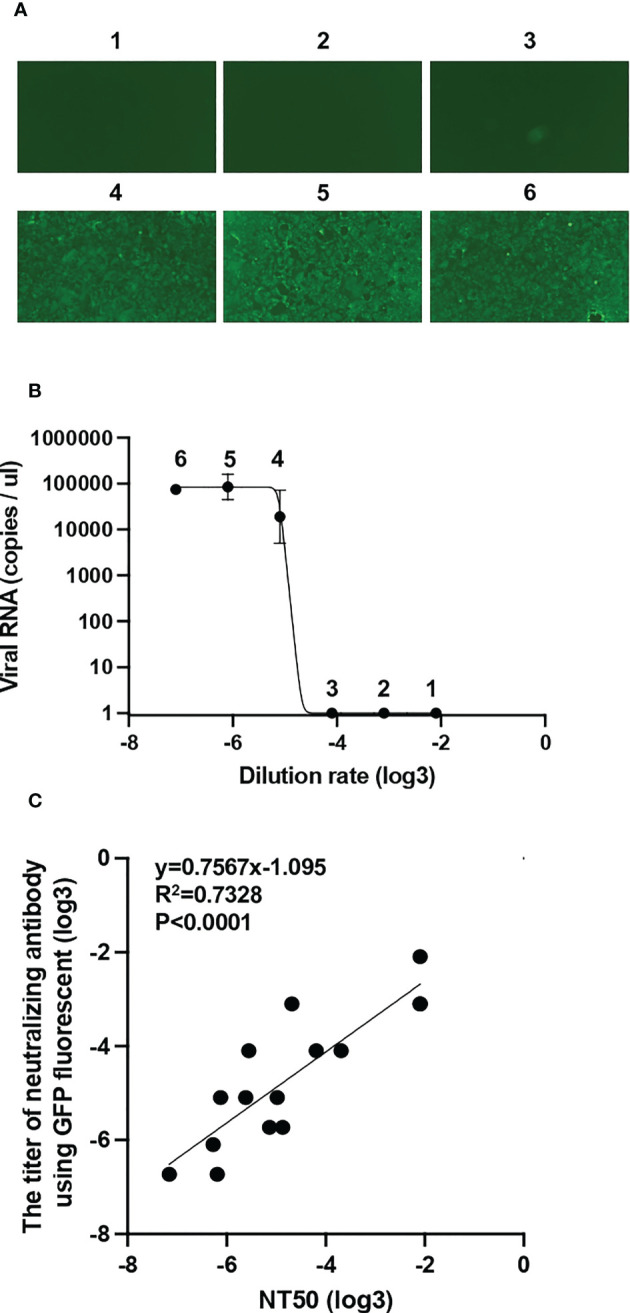
Establishment of a simplified system for assessing neutralizing activity by GFP detection **(A)** The mixture of diluted vaccinated sera and superfolder GFP-carrying SARS-CoV-2 was inoculated into VeroE6/TMPRSS2 cells. After 34 h post-infection, GFP fluorescence intensity was observed and scored by fluorescence microscopy: 1, 1:10; 2, 1:30; 3, 1:90; 4, 1:270; 5, 1:810; and 6, 1:2430 dilutions. **(B)** Viral RNA extracted from the supernatant of VeroE6/TMPRSS2 cells inoculated with diluted sera and recombinant SARS-CoV-2 mixture was quantified by RT-qPCR. **(C)** Correlation between 50% neutralization titers (NT_50_) measured by RT-qPCR and neutralizing antibody titer measured by GFP signal from 15 vaccinated individuals. A simple linear regression model was used to calculate correlation coefficient (R2) and the two-tailed p value.

To measure the neutralizing activity of sera, we developed a high-throughput neutralizing activity evaluation system. We previously generated a superfolder GFP (sfGFP)-carrying recombinant SARS-CoV-2 using an innovative reverse genetics method (**Figure S1A**) (Torii et al., 2021). This recombinant SARS-CoV-2 was mixed with diluted vaccinated serum and inoculated into the VeroE6/TMPRSS2 cells. The expression of GFP in VeroE6/TMPRSS2 cells was observed by fluorescent microscopy at 34 hpi. The percentage of cells expressing GFP increased with increasing serum dilution rate (**Figure 2A**). Simultaneously, we collected the supernatants of VeroE6/TMPRSS2 cells and quantified viral RNA using RT-qPCR. Similar to the GFP detection system, the quantity of viral RNA also increased at a high serum dilution rate (**Figure 2B**). To confirm the effectiveness of the neutralization assay by GFP detection, we compared NT_50_ calculated by RT-qPCR and neutralizing activity titer calculated by GFP detection (see Methods) in 15 vaccinated serum samples. NT_50_ were found to be well correlated with the titer of neutralizing antibody using GFP fluorescent (**Figure 2C**). These results suggest that the simplified method established in this study can be used for neutralization assays.

**Figure 3:**
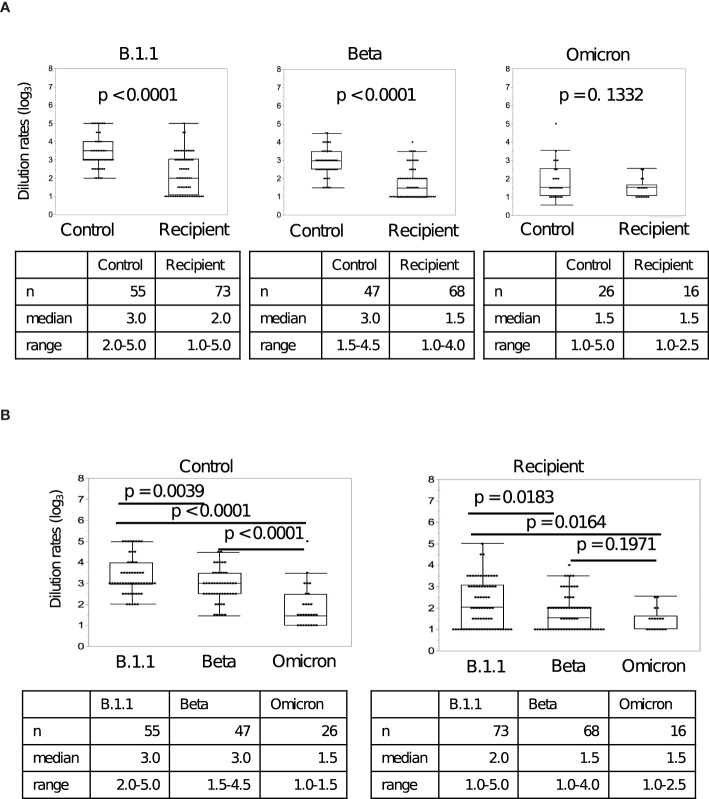
Neutralizing activity of sera against SARS-CoV-2 variants after the second vaccine dose. **(A)** The differences in serum dilution rates of living-donor liver transplant (LDLT) recipients and healthy controls against the Ancestral, Beta, and Omicron variants. **(B)** The differences in serum dilution rates of LDLT recipients and healthy controls between the Ancestral, Beta, Omicron variants.

The neutralizing activities of sera from controls and LDLT recipients against rB.1.1 S-GFP, rBeta S-GFP, and rBA.1 S-GFP were examined. As shown in **Figure 3A**, the dilution rates of the recipient sera were approximately 3-fold lower against the Ancestral strain and approximately 5-fold lower against the Beta strain than those of the control sera. However, the dilution rate against the Omicron strains was comparable between the two groups. As shown in **Figure 3B**, the dilution rates of the control and recipient sera against rBeta S-GFP and rBA.1 S-GFP were significantly lower than those against rB.1.1 S-GFP. These results suggest that after the second vaccine dose, the dilution rates against the Ancestral and Beta variants were low in recipient sera compared to those in control sera; however, the dilution rate against the Omicron variant was null in both groups.

In addition, we examined the correlation between the comparative absorbance value and the dilution rates. In **Figure S2**, there were significant correlations in Ancestral and Beta strains, while there was no significant correlation in Omicron strain.

### 3.3 Neutralizing activity of sera against SARS-CoV-2 variants after third vaccine dose

**Figure 4:**
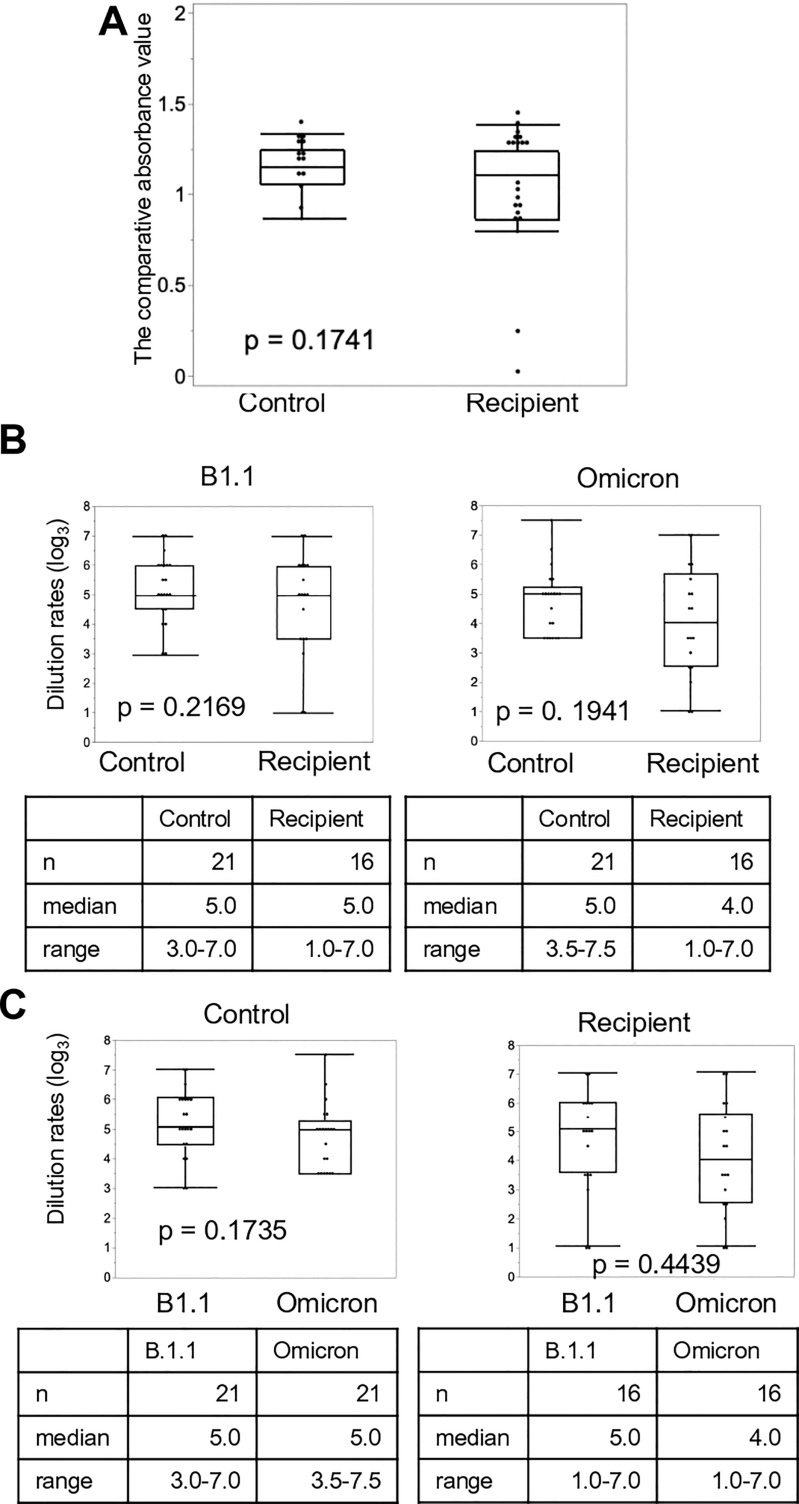
Neutralizing activity of sera against SARS-CoV-2 variants after the third vaccine dose. **(A)** Anti-SARS-CoV-2 spike protein antibodies in living-donor liver transplant (LDLT) recipients and healthy controls were measured using ELISA. **(B)** The differences in serum dilution rates of recipients and healthy controls against the Ancestral and Omicron variants. **(C)** The differences in serum dilution rates of LDLT recipients and healthy controls between the Ancestral, and Omicron variants.

We investigated the titers of neutralizing antibodies using ELISA. The titer after the third vaccine dose was elevated in both controls and LDLT recipients (**Figure 4A**). The increase in the median neutralizing antibodies titer from the second to the third vaccine dose was approximately 1.2-fold and more than 2-fold in the controls and LDLT recipients, respectively. Furthermore, approximately 25% of LDLT recipients had no neutralizing antibodies after the second dose, but the rate decreased to only about 5% after the third dose. Next, we evaluated the dilution rates of the control and recipient sera against rB.1.1 S-GFP and rBA.1 S-GFP after the third vaccine dose (**Figure 4B**). No significant difference was observed in the dilution rates against rB.1.1 S-GFP between the controls and LDLT recipients. Additionally, the dilution rate against rBA.1 S-GFP was comparable to that against rB.1.1 S-GFP in both groups (**Figure 4C**).

In addition, we examined the correlation between the comparative absorbance value and the dilution rates. In **Figure S3**, there were significant correlations in both Ancestral and Omicron strains after third vaccination.

## 4 Discussion

The response to the second vaccine dose was quite different between the controls and LDLT recipients, and the neutralizing activity against SARS-CoV-2 variants after the second dose was markedly low in the LDLT recipients. However, after the third dose, the titer of neutralizing antibodies increased, especially in the recipients, and the difference between the two groups in the neutralizing activity of the serum against the variants disappeared.

In the current study, the neutralizing antibody titer in recipient sera after the second vaccination was approximately half than in the control sera, which is consistent with the results by Rabinowich et al. (Rabinowich et al., 2021). We showed that predictors of non-responders among LDLT recipients were higher age at vaccination, presence of ascites at vaccination, multiple immunosuppresant treatment, and high MMF dose. Several risk factors for non-responders with solid organ transplantation, including LT, after the second vaccine dose, have been previously reported. Higher age at vaccination was reported to deteriorate vaccine efficiency in LT(Rabinowich et al., 2021), consistent with other preliminary reports regarding the effect of older age on vaccine response in non-immunosuppressant people (Müller et al., 2021) and solid organ recipients (Boyarsky et al., 2021). The presence of ascites at vaccination correlated with non-response after the second vaccination (Toniutto et al., 2022a). Liver dysfunction has been reported to deteriorate vaccine efficacy (Thuluvath et al., 2021), and post-transplant liver function may also be an important factor in vaccine efficacy. Immunosuppressive treatment with MMF has been associated with deteriorating SARS-CoV-2 antibody responsees in heart (Peled et al., 2021) and liver transplantations (Rabinowich et al., 2021; Toniutto et al., 2022). Multiple immunosuppressive treatments may also be a risk factor due to MMF use (Grupper et al., 2021; Peled et al., 2021; Rabinowich et al., 2021; Toniutto et al., 2022). Calcineurin inhibitors, including tacrolimus and cyclosporine, are the principal immunosuppressants. While the amount and concentration of tacrolimus itself is not a risk factor for non-responders, high amounts of calcineurin inhibitors increase the risk of deteriorating kidney function. If the recipient’s renal function is impaired, the calcineurin inhibitor dose should be reduced, and everolimus or other combination therapies should be considered for renal protection. A high MMF dose was also a risk factor for non-responders, and the dose was correlated with neutralizing antibody titer. In LDLT, blood type-incompatible transplants are more common than in deceased-donor liver transplantation, and high MMF doses tend to be used. Therefore, when blood type-incompatible transplantation is performed, vaccination before transplantation should be considered, or if the vaccine is administered after transplantation, MMF dose reduction should be taken into account whenever possible.

This study showed that the third vaccine dose was very effective, even in the recipients; the rate of responders among recipients increased from 75% to 95% after the third dose. Although the immune response rate after the second vaccine dose was low among solid organ transplant recipients, (Grupper et al., 2021; Korth et al., 2021; Peled et al., 2021) it was relatively high and reported to be 47.5–79% in LT recipients (Rabinowich et al., 2021; Rashidi-Alavijeh et al., 2021; Davidov et al., 2022; Ruether et al., 2022). Although the usefulness of the third dose in LT recipients has not yet been reported, Davidov et al. (Davidov et al., 2022) reported that the percentage of RBD-binding IgG immune responses after the second dose (56%) improved significantly after the third dose (98%). We also found that the immune response rates after the third dose increased considerably in LDLT recipients, reaching approximately the equivalent level achieved by the control (Davidov et al., 2022). Whether the fourth SARS-CoV-2 mRNA vaccine dose is effective in LT recipients who tested negative after the third dose or whether the vaccine type should be changed is a question to be studied in the future. In addition, Omicron dilution rates and the comparative absorbance value did not show a significant correlation after the second vaccination, but did show a significant correlation after the third vaccination. This suggests that the third dose may bring qualitative changes in antibodies.

In LT recipients, although quantitative changes in neutralizing antibody titers have been studied, to the best of our knowledge, no such studies have been conducted to assess neutralizing activity of vaccine-induced antibody against SARS-CoV-2 variants. Experiments with SARS-CoV-2 pseudoviruses have confirmed that two vaccine doses induce low neutralizing activity against SARS-CoV-2 variants, especially the Beta and Omicron variants. Moreover, the neutralizing activity of sera against the Omicron variant appeared after the third vaccine dose in healthy controls; however, vaccine efficiency after the third dose against various SARS-CoV-2 variants in LT recipients is unknown (Rabinowich et al., 2021). Therefore, we evaluated the neutralizing activities of LT recipient sera using recombinant chimeric SARS-CoV-2 with spikes replaced by Beta and Omicron spikes. In healthy control sera, the neutralizing activities against the Beta and Omicron variants were lower than those against the Ancestral strain after the second vaccination; however, (Muik et al., 2022) the neutralizing activities against the Beta and Omicron variants were also reduced in recipient sera. Especially, recipients showed no neutralizing activities against the Omicron variant, although controls also had low neutralizing activities. However, the third vaccine dose increases the neutralizing activities against the variants (Muik et al., 2022). In this study, recipient sera as well as healthy control sera showed an increase in neutralizing activities against the Beta and Omicron variants to levels comparable to that of the Ancestral strain. Currently, the Omicron variant predominates, and quantitative evaluation of antibodies against each prevalent variant is necessary in future studies.

This study had some limitations. First, the time between vaccination and blood collection was varied because the blood samples were collected during the outpatient visit. Although LDLT recipient had shorter term than controls, LDLT recipients had lower neutralizing activity. Therefore, the timing of sample collection did not seem to have significant effect on current results. Second, a relatively larger number of samples (nearly 150) were collected after the second vaccination than after the third dose, warranting further investigation.

In conclusion, only the second vaccine dose was not effective in LDLT recipients, and the third dose was as effective as that in healthy controls, with sufficient neutralizing activities against SARS-CoV-2 variants. Moreover, we established a new simplified assay for assessing antibody neutralizing activity by GFP detection.

## Data availability statement

The raw data supporting the conclusions of this article will be made available by the authors, without undue reservation.

## Ethics statement

The studies involving human participants were reviewed and approved by the Institutional Review Board of Kyushu University Hospital (approval number 2020-639). Written informed consent for participation was not required for this study in accordance with the national legislation and the institutional requirements.

## Author contributions

Conception and design: TTomiy, RS, NH, TY, and TF. Methodology development: TTomiy, RS, TTa, HI, KTo, NK and TF. Data acquisition: TTomiy, RS, TTa, NH, SI, KTo, Y-K-F, TTomin, KTa, ShY, YN, TY, and TF. Data analysis and interpretation: TTomiy, RS, NH, SI, SaY, TK, TY, and TF. Writing and/or revision of manuscript: TTomiy, RS, TY, and TF. Study supervision: TY and TF. All authors contributed to the article and approved the submitted version.
