# Supplementary material for: A third dose of the BNT162b2 mRNA vaccine sufficiently improves the neutralizing activity against SARS-CoV-2 variants in liver transplant recipients
Source: Front Cell Infect Microbiol. 2023 May 16;13:1197349. doi: 10.3389/fcimb.2023.1197349 (PMC10229048; doi:10.3389/fcimb.2023.1197349)
Supplement: Supplementary file 1 [file DataSheet_1.pdf]

Figure.S1

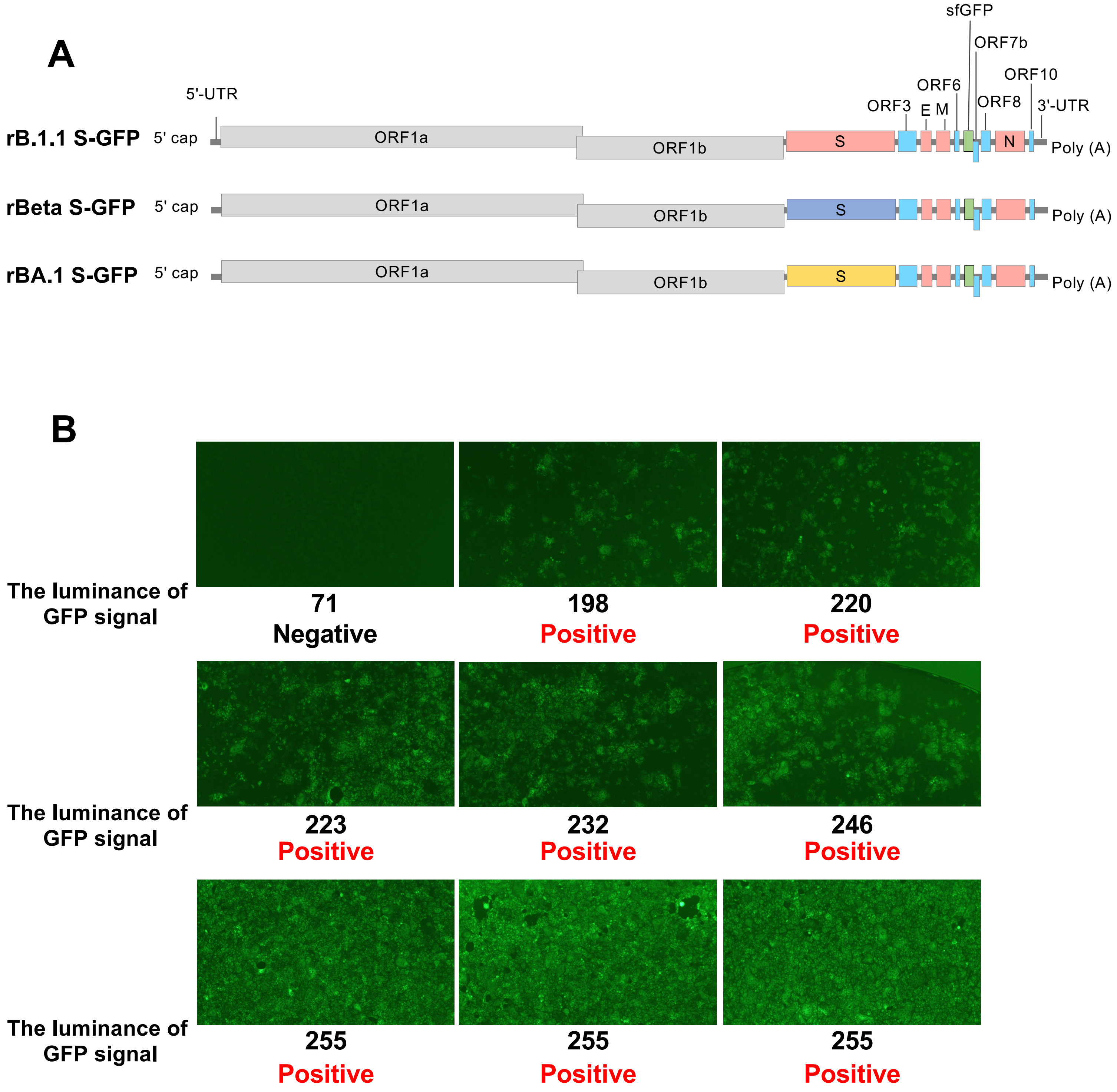

Figure S1 legends

(A) Scheme for the chimeric recombinant SARS-CoV-2 used in this study. The SARS-CoV-2 genome and its genes are shown. *ORF7a* was swapped with the *sfGFP* gene. (B) Fluorescent foci of sfGFP carrying recombinant SARS-CoV-2 on VeroE6/TMPRSS2 cells. Original images were collected by fluorescent microscopy and the luminance of GFP was calculated by Image J. The calculation of a titer of neutralizing antibody is described in Methods.

Figure.S2

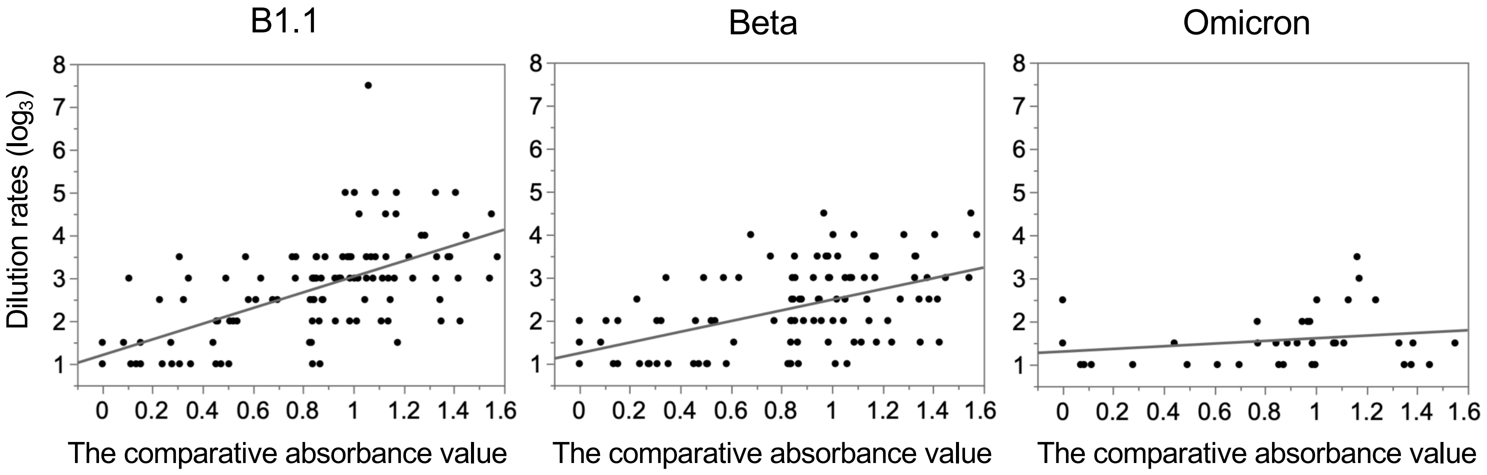

$r^2 = 0.437044$
$p < 0.0001$

$r^2 = 0.315898$
$p < 0.0001$

$r^2 = 0.043969$
$p = 0.2129$

Figure S2 legends

The examination with the comparative absorbance value in ELISA and dilution rate in Ancestral, Beta and Omicron strains.

Figure.S3

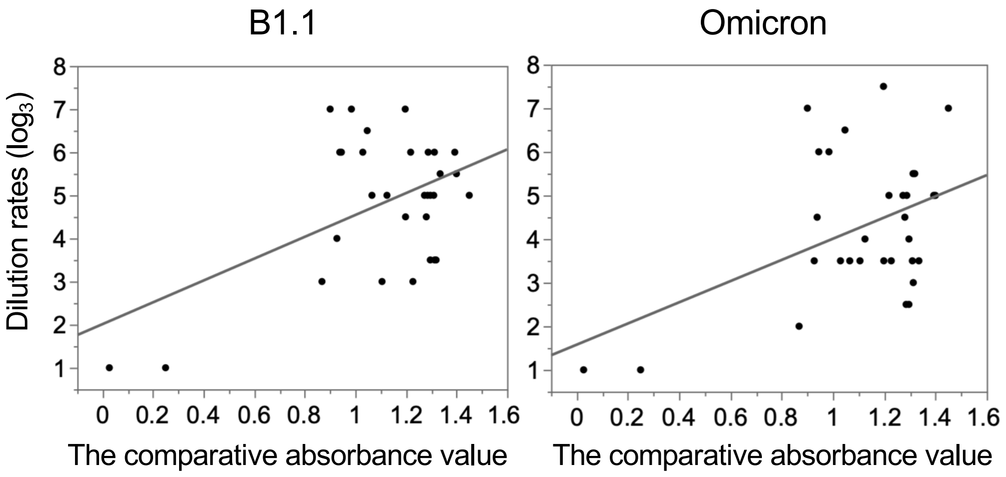

$r^2 = 0.250536$
$p = 0.0041$

$r^2 = 0.315898$
$p < 0.0001$

Figure S3 legends

The examination with the comparative absorbance value in ELISA and dilution rate in Ancestral, and Omicron strains.
