# Supplementary material for: A third dose of the BNT162b2 mRNA vaccine sufficiently improves the neutralizing activity against SARS-CoV-2 variants in liver transplant recipients
Source: Front Cell Infect Microbiol. 2023 May 16;13:1197349. doi: 10.3389/fcimb.2023.1197349 (PMC10229048; doi:10.3389/fcimb.2023.1197349)
Supplement: Supplementary file 2 [file Table_1.docx]

| **Table S1. Primers and proves used in this study** | |  |
| --- | --- | --- |
| Primer name | Primer sequence (5'-to-3') | Purpose |
| Linker_F1-Fw | CTATATAAGCAGAGCTCGTTTAGTGAACCGTATTAAAGGTTTATACCTTCCCAGGTAAC | CPER fragment 1 preparation |
| F1-F2-Rv | CAGATTCAACTTGCATGGCATTGTTAGTAGCCTTATTTAAGGCTCCTGC | CPER fragment 1 preparation |
| F1-F2-Fw | GCAGGAGCCTTAAATAAGGCTACTAACAATGCCATGCAAGTTGAATCTG | CPER fragment 2 preparation |
| F2-F3-Rv | GGTAGGATTTTCCACTACTTCTTCAGAGACTGGTTTTAGATCTTCGCAGGC | CPER fragment 2 preparation |
| F2-F3-Fw | GCCTGCGAAGATCTAAAACCAGTCTCTGAAGAAGTAGTGGAAAATCCTACC | CPER fragment 3 preparation |
| F3-F4-Rv | GGTGCACAGCGCAGCTTCTTCAAAAGTACTAAAGG | CPER fragment 3 preparation |
| F3-F4-Fw | CACCACTAATTCAACCTATTGGTGCTTTGGACATATCAGCATCTATAGTAGCTGGTGG | CPER fragment 4 preparation |
| F4-F5-Rv | GTTTAAAAACGATTGTGCATCAGCTGACTG | CPER fragment 4 preparation |
| F4-F5-Fw | CACAGTCTGTACCGTCTGCGGTATGTGGAAAGGTTATGGCTGTAGTTGTGATC | CPER fragment 5 preparation |
| F5-F6-Rv | GCGGTGTGTACATAGCCTCATAAAACTCAGGTTCCCAATACCTTGAAGTG | CPER fragment 5 preparation |
| F5-F6-Fw | CACTTCAAGGTATTGGGAACCTGAGTTTTATGAGGCTATGTACACACCGC | CPER fragment 6 preparation |
| F6-F7-Rv | CATACAAACTGCCACCATCACAACCAGGCAAGTTAAGGTTAGATAGCACTCTAG | CPER fragment 6 preparation |
| F6-F7-Fw | CTAGAGTGCTATCTAACCTTAACTTGCCTGGTTGTGATGGTGGCAGTTTGTATG | CPER fragment 7 preparation |
| F7-F8-Rv | CTAGAGACTAGTGGCAATAAAACAAGAAAAACAAACATTGTTCGTTTAGTTGTTAAC | CPER fragment 7 preparation |
| F7-F8-Fw | GTTAACAACTAAACGAACAATGTTTGTTTTTCTTGTTTTATTGCCACTAGTCTCTAG | CPER fragment 8 preparation |
| F8-F9-Rv | GCAGCAGGATCCACAAGAACAACAGCCCTTGAGACAACTACAGCAACTGG | CPER fragment 8 preparation |
| F8-F9-Fw | CCAGTTGCTGTAGTTGTCTCAAGGGCTGTTGTTCTTGTGGATCCTGCTGC | CPER fragment 9 GFP preparation |
| F9-Linker-Rv | GGAGATGCCATGCCGACCCTTTTTTTTTTTTTTTTTTTTTTTTTGTCATTCTCCTAAG | CPER fragment 9 GFP preparation |
| F9-Linker-Fw | CTTAGGAGAATGACAAAAAAAAAAAAAAAAAAAAAAAAAGGGTCGGCATGGCATCTCC | CPER fragment 9 Linker preparation |
| Linker_F1-Rv | GTTACCTGGGAAGGTATAAACCTTTAATACGGTTCACTAAACGAGCTCTGCTTATATAG | CPER fragment 9 Linker preparation |
| covid-dp-Beta fr8-Fw | TGCCTGCAGGTCGACATGTTTGTTTTTCTTGTTTTATTGCCACTAG | Beta CPER F8 fragment preparation |
| covid-dp-Beta fr8-Rv | ACCTCTAGACTCGAGAAGTTCGTTTATGTGTAATGTAATTTGACTCC | Beta CPER F8 fragment preparation |
| RT-qPCR N protein F | CACATTGGCACCCGCAATC | RT-qPCR preparation |
| RT-qPCR N protein R | GAGGAACGAGAAGAGGCTTG | RT-qPCR preparation |
| RT-qPCR N protein probe | FAM-ACTTCCTCAAGGAACAACATTGCCA-BHQ | RT-qPCR preparation |
